# Using Codesign to Develop a Novel Oral Healthcare Educational Intervention for Undergraduate Nursing Students

**DOI:** 10.3390/ijerph20064919

**Published:** 2023-03-10

**Authors:** Jacqueline Rojo, Ajesh George, Yenna Salamonson, Leanne Hunt, Lucie M. Ramjan

**Affiliations:** 1 School of Nursing and Midwifery, Western Sydney University, Locked Bag 1797, Penrith, NSW 2751, Australia; 2 Australian Centre for Integration of Oral Health (ACIOH), School of Nursing and Midwifery, Western Sydney University, Ingham Institute for Applied Medical Research, Locked Bag 7103, Liverpool, NSW 2170, Australia; 3 School of Dentistry, Faculty of Medicine and Health, The University of Sydney, Sydney, NSW 2006, Australia; 4 School of Nursing, University of Wollongong, Wollongong, NSW 2522, Australia

**Keywords:** codesign, intervention, undergraduate nursing, education, oral healthcare, Mezirow’s Transformative Learning theory

## Abstract

To build a nursing workforce that is equipped to undertake oral health promotion and screening, an educational program was needed. With codesign being used in multiple settings, it was selected as the approach to use, with Mezirow’s Transformative Learning theory as the underpinning framework. This study aimed to develop an oral healthcare educational intervention for nursing students. Using a six-step codesign framework, nursing students and faculty staff were invited to participate in two Zoom™ Video Communication workshops to codesign the learning activities to be used in the classroom. The codesign process was evaluated through focus groups and analysed using a hybrid content analysis approach. A multifaceted oral healthcare educational intervention was developed. Learning material was delivered using a range of different learning and teaching resources such as dental models, podcasts, and an oral health assessment across two subjects. Multiple approaches to recruitment, the inclusion of participants, and good facilitation of workshop discussions were critical to the codesign of the educational intervention. Evaluation revealed that preparing participants prior to the workshops acted as a catalyst for conversations, which facilitated the codesign process. Codesign was a useful approach to employ in the development of an oral healthcare intervention to address an area of need.

## 1. Introduction

The World Health Organization reports that approximately 3.5 billion people worldwide are affected by oral disease, and the financial burden of oral disease has been estimated to equal almost $390 billion USD [1]. In addition to the financial burden, oral disease impacts an individual’s systemic health [2]. Traditionally, oral disease is managed by dental professionals; however, with increasing prevalence, guidelines have been developed to expand the roles of other health professionals, such as nurses to screen for dental disease for early intervention [3].

To build a future workforce that is well equipped to undertake this role, the development of an educational program for nursing students is needed. While there have been reports of the integration of oral health programs in the United States and isolated reports in Canada, the United Kingdom, Norway, and Brazil, the integration of oral healthcare education in Australia has been reported only in midwifery education [3]. An oral healthcare educational intervention for Australian nursing students is, therefore, required. Such a program would enable students to take preventative action against oral disease in practice settings, reducing the disease burden that poor oral health presents to the population.

To develop an oral healthcare educational intervention, a codesign approach has the potential to produce an intervention that is sustainable and translational in improving healthcare delivery. Codesign uses creative and collaborative methods of participation between consumers and service providers [4]. It has been used in industries such as computing [5] and healthcare [6,7] to improve service delivery. The approach has gained popularity in higher education, such as universities, for designing courses [8,9]. In course design, feedback garnered from learners as well as teachers is incorporated in the development of class activities, which facilitates course design [10]. Through a codesign approach, learning material is co-created *with* students, rather than *for* students, who will directly benefit from engagement with the learning content [11]. This approach provides a unique perspective which moves away from conventional course designs. For example, current design practices in the school where this study is based are largely teacher-focused. The codesign framework enables the shift away from this focus to a student-centred approach, whereby the learners inform the designers how best they learn. This has the potential to further promote the success of the program.

While there are a number of codesign frameworks that can be utilised to undertake codesign research [9,10,12], the framework by Dietrich, Trischler, Schuster, and Rundle-Thiele [10] is a useful step-by-step guide that enables mutually respectful partnerships to be developed between participants exposed to a power-imbalance (students) and faculty staff. Inclusion of students in the design process enables the student voice to be heard and promotes student engagement [9], as both students and the faculty participate in robust discussions to decide on the most effective approach to teaching in the classroom setting. Another example from Gros and López [9] is that the authors adopted a codesign approach in higher education, with students supporting the design of learning activities and selecting the digital resources to be used during the design of multiple courses. However, the model that was used did not provide a step-by-step outline, as was the case for the model by Dietrich, Trischler, Schuster, and Rundle-Thiele [10], on how to conduct the codesign process, from planning to recruitment and facilitation of workshops. Furthermore, the model used by Gros and Lopez [9] indicates that students and teachers were separated, thereby not enabling partnerships to be built.

Limited research formally documents the codesign process. This study is, to our knowledge, the first to comprehensively report on the development of an oral healthcare intervention for undergraduate nursing students, by using the codesign framework espoused by Dietrich, Trischler, Schuster, and Rundle-Thiele [10] and evaluating the process.

## 2. Materials and Methods

### 2.1. Research Context

This study forms a part of a larger mixed-methods project aimed at integrating oral healthcare into the undergraduate Bachelor of Nursing (BN) curriculum at a large, multi-campus Australian university. The BN is a three-year preregistration nursing program with limited oral healthcare education. This paper comprehensively reports on the process of developing the oral healthcare intervention using the six-step codesign framework of Dietrich, Trischler, Schuster, and Rundle-Thiele [10] within the methods and evaluates the process.

### 2.2. Participants

The participants in the codesign study were second-year undergraduate nursing students and faculty staff involved in the teaching of a second-year chronic illness theory subject. Those teaching in the concurrent practical (clinical) subject were also included in the study.

### 2.3. Codesign Framework and Theory Underpinning the Codesign Process

**Figure 1:**
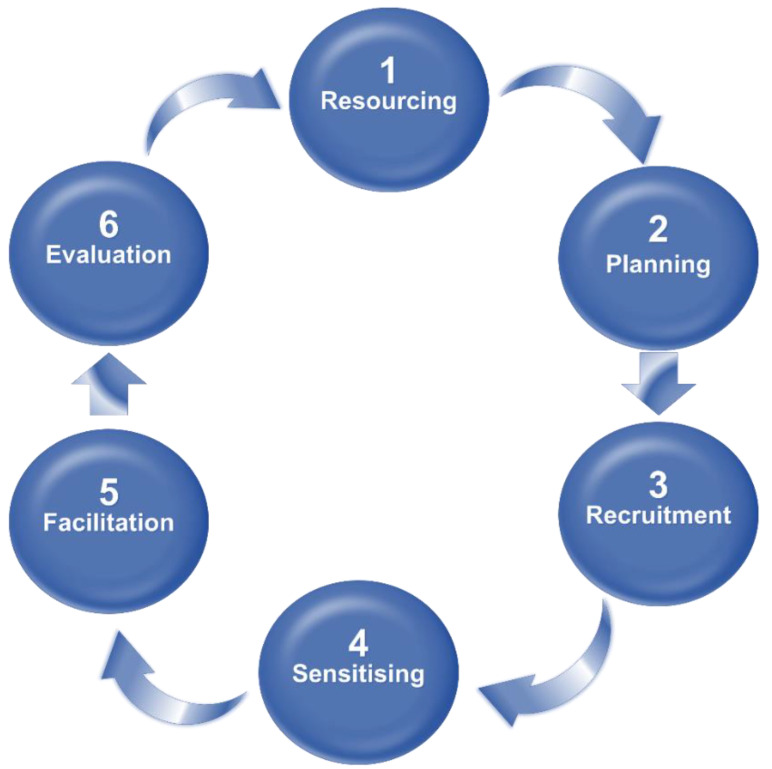
Six-step process of codesign Dietrich, Trischler, Schuster, and Rundle-Thiele [10].

The six-step process of codesign Dietrich, Trischler, Schuster, and Rundle-Thiele [10], which is outlined in Figure 1, was the framework used to develop the teaching and learning activities for the oral healthcare intervention. This framework was selected because students are vulnerable consumers in education. As the intervention also required a theoretical underpinning that would allow students to transform their perspectives, Mezirow’s Transformative Learning theory was selected for this study. This 7-phase theory enables learners to change and transform their mindset about a given topic [13]. In this case, transforming mindsets on oral healthcare may be required to transform current attitudes towards oral healthcare in nursing.

### 2.4. Application of Dietrich’s Six-Step Process of Codesign in the Development of an Oral Healthcare Educational Intervention for Nursing Students

#### 2.4.1. Step One—Resourcing

This first step is designed to enable the researcher to ascertain the resources that would be needed for the codesign workshops and that would enhance understanding of the problem and promote participation. To facilitate this step in this study, two robust literature reviews were conducted to identify which setting best enabled learning. Furthermore, activities which best aligned with Mezirow’s Transformative Learning Theory [3,13] were identified. From the first review, it was identified that developing the education across more than one subject achieved better learning outcomes, such as increased knowledge and confidence with oral healthcare [3]. Based on this information, the researchers specifically targeted a second-year theoretical chronic illness subject and a concurrent second-year practical subject for the study. The second review identified transformative learning activities [13] which were good learning strategies to use to achieve transformation. These included group learning; experiential activities, such as simulation; and reflective activities.

#### 2.4.2. Step Two—Planning

This step includes planning and preparation for the codesign sessions and was conducted by the researcher in collaboration with the research team and research assistant (RA). For this study, it was decided that two one-hour sessions would be conducted one week apart from each other. This was to enable participants to present other activities and ideas that they had not considered during the first workshops. The first session would be the codesign workshop, in which students and faculty participants would engage in the design of the learning activities. Additionally, as the oral healthcare educational intervention was focused on integrating oral healthcare in the undergraduate nursing curriculum, the team decided to source industry experts from aged care and intensive care to attend the codesign workshop. The industry experts would present the current oral healthcare practices in their respective settings for 10 min at the commencement of the workshop, providing context for the participants.

The second session would be an evaluation session to evaluate the codesign process. Due to the large multisite nature of the university where the research was undertaken and the COVID-19 restrictions in place at the time, a decision was made among the team to conduct the codesign workshops online via Zoom™ Video Communications, which allowed greater flexibility for participants to attend.

#### 2.4.3. Step Three—Recruitment

This third step involves the recruitment of participants. The sample included staff teaching in the theoretical and practical subjects, as well as second-year nursing students. At the time of the research, students had not yet undertaken the subjects in which the educational intervention was to be embedded. Convenience sampling was undertaken, and an email was sent to both staff and students informing them of the codesign workshops and inviting them to participate. The email detailed the purpose of the workshops, which was to codesign the learning activities which would be used to deliver the oral health education. A student RA also explained the study in a 1 min recruitment video (https://youtu.be/qeBbh9HgBQo, accessed on 13 February 2023). This video was placed on students’ Blackboard™ learning sites (digital learning platform). Students and staff that were interested in participating contacted the RA via email, and the consent process took place. Any additional queries that potential participants had were answered at this time. An electronic poll was also sent to participants which provided a range of workshop times; this provided greater options for the participants. Once the final workshop times were organised, calendar invites were sent to all the participants.

#### 2.4.4. Step Four—Sensitising

This step relates to the researcher preparing the participants for what is to come, thereby introducing them to the topic for discussion. For this step, a resource booklet was developed (see Supplementary Document S1) that was emailed to the participants prior to the first workshop. The booklet provided details of the concept of codesign and explained the learning theory—Mezirow’s Transformative Learning Theory—that was to be used. Based on Step One learnings, the resource provided guidance on the evidence-based educational activities that the participants could select. Activities included the 1 min essay, oral health assessment, and reverse case study. Participants were also encouraged to present their own ideas at the workshops. Reminder emails about the workshops were sent three times prior to the workshops, as well as the day before.

#### 2.4.5. Step Five—Facilitation

This step includes the facilitation of the codesign workshops. For Workshop 1, students and staff were separated into two groups which included a mix of students and faculty staff. One group was facilitated by the RA, and the other was facilitated by the researcher. The first workshop ran overtime at 1 h and 30 min. In these groups, discussions were had regarding the learning activities, which included the dental models, podcasts, and oral health assessment. Other innovative ideas generated by students were the use of animations, videologs (vlogs), and videocasts (vodcasts). The learning activity discussion followed the phases of Mezirow’s learning theory, to ensure that there was an activity addressing each phase. Participants were encouraged to discuss which learning activity was suitable for each phase of the learning theory based on the resource which was provided to them in Step Four.

During Workshop 2, each group presented their codesigned learning activities for five minutes each, and the researcher facilitated the discussion, which compared the activities that had been selected by each of the groups. The comparison was then used to evaluate the process in Step Six. At the completion of these discussions, participants were then divided into staff- and student-only focus groups, and an evaluation of the codesign process was conducted.

#### 2.4.6. Step Six—Evaluation

This final step enabled the researcher to evaluate the data from the workshops and design a plan of action. In this step, after the participant groups had presented their ideas in Workshop 2 (Step Five), a discussion was held between all participants, and a final sequence of activities was decided. The suggestions made by participants were collated, and the final activity series was set based on alignment to the seven phases of Mezirow’s Transformative Learning theory (Table 1). At the completion of this step, the researcher organised a meeting with the Subject Coordinators of the theoretical and practical subjects to present the plan generated in the codesign process. The Subject Coordinators had been approached prior to the workshops and had agreed for this process to occur. Other prior agreements were obtained from the Deputy Dean, Associate Dean for Learning and Teaching, and Director of Academic Program. The researcher then developed the resources required, made changes to the student guides for the classes, purchased the models, and organised the subject-matter-expert-led podcasts to be recorded. These processes occurred four to five weeks before the teaching semester commenced.

### 2.5. The Oral Healthcare Intervention

**Figure 2:**
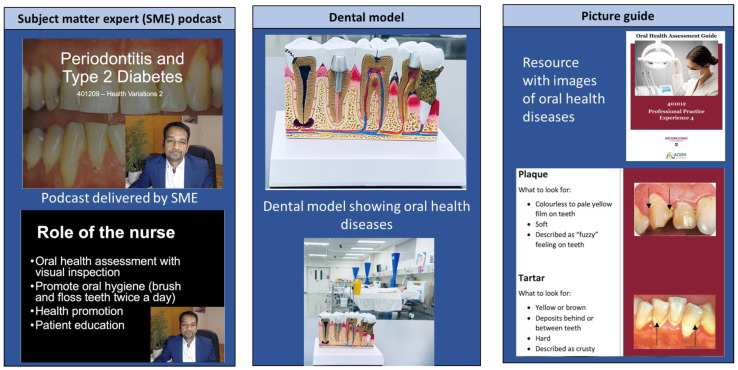
Example learning activities.

Based on the completion of the six steps, the resultant oral healthcare intervention included the use of a case study without diagnosis in the theoretical subject, followed by a self-reflection activity and whole-class discussion (see Supplementary Document S1). Subject-matter-expert-led podcasts were created based on the theoretical aspects of periodontitis and type 2 diabetes mellitus due to the bidirectional relationship of these conditions, with periodontal treatment improving glycaemic control. The selection of this content was part of a suite of education to be delivered to undergraduate nursing students across multiple subjects. Prior to the education being delivered in this chronic disease subject, the nursing students had completed oral healthcare training in a first-year primary healthcare subject. These podcasts were made available on the Blackboard learning site. Practice quiz questions as knowledge checks were also included. For the practical subject, teaching and learning activities included the use of dental models, a video or tutor demonstration of an oral health assessment and simulated practice of an oral health assessment. The final resource that was developed as a learning activity in the practical subject was a picture guide of the different dental pathologies included in the oral health assessment, such as caries and receding gums (Table 1 and Figure 2).

### 2.6. Data Analysis

Focus group evaluation data from the second workshop was analysed to explore the codesign process. A hybrid approach was adopted that enabled both deductive and inductive analysis, as described by Elo and Kyngäs [14] and conducted by Bray et al. [15]. This approach allowed for data to be organised and understood by researchers in a meaningful manner. The analysis was conducted in three stages: (1) Preparation; (2) Organisation; and (3) Reporting [14]. In stage 1, the researchers immersed themselves in the data to obtain an overall understanding of the whole codesign experience. In the second stage, using a deductive approach, a categorisation matrix using five of the six steps of Dietrich’s codesign process was conducted. Data were then coded into each respective category. Following this, using an inductive approach, data were recoded and recategorised into sub-categories. The third stage of the analysis, which involves the reporting of findings, is included in detail in the evaluation section of this paper.

### 2.7. Ethical Considerations

Ethical approval for this study was received from the Western Sydney University Human Research and Ethics Committee (H14177). Participation in this study was voluntary, and consent was obtained prior to the workshops and confirmed at the beginning of each workshop and focus group (which were recorded). Participants were assigned a pseudonym, and the transcripts were deidentified to maintain confidentiality. All participants received a certificate of participation in appreciation of their time and effort.

## 3. Results—Evaluation of the Codesign Process

### 3.1. Participants

Ten participants were scheduled to attend the workshops; however, a total of eight participants attended: five academic staff members and three second-year nursing students. All eight participants were female, and all contributed throughout the workshops. Of the academic staff members, four were permanent, and one was casual. Of the two students that did not attend, one attended the second workshop only but did not remain for the evaluation. A second student withdrew on the day of the workshop due to other commitments.

### 3.2. Evaluation of the Codesign Process

Categorisation of data occurred in alignment with the first five stages of the codesign framework used in this study. The process is summarised in Table 2.

#### 3.2.1. Category 1—Resourcing

As the learning activities were the focus of the resourcing stage, this was what dominated the discussion. Providing numerous learning activity options in the booklet that was developed assisted in preparing the participants prior to attending Workshop 1. Examples of these activities included the oral health assessment, models, and videos. These options enabled the generation of discussions and prompted “new” ideas.

##### Learning Activities—“Catalyst to Make Me Start Thinking”

Both staff and students agreed that the learning activities that were presented to them acted as catalysts for discussion within their groups, which prompted further ideas to be considered. Discussions may not have been as robust had the number of activities available not been sufficient, which emphasises the importance of appropriate resourcing.

*“They [learning activities] gave you good prompts for developing them [learning activities] further…. I think within our group, we did a lot of that. Like “oh, how about we do this instead?”*

[Staff 1]

*“The learning activities that [organiser] provided were really good…. from reading…. her learning activities, it gave me that catalyst to like make me start thinking of my other learning activities ‘cause it was just like a point she gave and I can continue on with it.”*

[Student 3]

##### Learning Activities—“Triggers That Critical Thinking”

Overall, participants were happy with the selection of activities provided to them; they felt that there was a good range and this variety also stimulated engagement as well as critical thinking processes. The stimulation of critical thinking is important in the learning process, particularly when aligning activities to Mezirow’s Transformative Learning Theory. The reverse case study was a popular point of discussion for both students and staff in that staff felt that this activity would pique critical thinking, while students felt it was a little confusing:

*“There’s lots of really good examples. Like, I really particularly like the reverse case study, um, you know, it’s not something that we get to see a lot, but it’s quite relevant and triggers that critical thinking component.”*

[Staff 2]

*“Um, there was one learning activity she put there that I was a bit confused about. Um, I think it was… it wasn’t case study without a diagnosis, but it was something else. I think it was called reverse case study?”*

[Student 3]

#### 3.2.2. Category 2—Planning

The planning of the workshops included the time allocation for the workshops, the use of Zoom™, and frequent reminders sent to participants regarding the workshops. During the planning phase, the original idea was to conduct the workshops face-to-face; however, due to the COVID-19 pandemic restrictions, these workshops were held on Zoom™.

##### Workshop Timing—“I Didn’t Realise We Went Over”

Although the workshops were scheduled for one hour, the participants were engaged in the discussions and did not appear concerned that the first workshop went overtime by 30 min. In this case, increasing the timing of the workshop to one and a half hours would have been acceptable, although this may vary depending on the workshop content.

*“It was good. We went over, but actually it was great. I didn’t realise we went over. Like, it wasn’t boring, it was actually very engaging and I thought, yeah, an hour, and hour and a half is actually quite doable.”*

[Staff 2]

*“It was, like, actively engaging. We were lost. Like, you know, we lost track of time and I was like ‘Oh, an hour and a half already has passed by’ and everybody was still talking which is really good”*

[Student 2]

There was only one staff member that provided feedback that they felt that the time allocated for the workshops was too long:

*“The time was marginally too long but this may have been different if the other participant turned up”*

[Staff 5]

##### Benefits of Zoom™—“Convenience Definitely”

The participants identified that convenience was one of the biggest benefits of using Zoom™ for codesign workshops, particularly when juggling multiple commitments, such as work. One student participant also expressed that Zoom™ made her feel comfortable to express her opinions freely and to use her voice.

*“I believe Zoom would have been the most desirable choice to hold the one-hour workshop due to its conveniences.”*

[Student 1]

*“You know, um, time factor. You know, like not, if I’m at work, that’s great and I can come in, but I if, you know, say I’m not working and I’m taking time to go to work particularly for a two-hour, you know, workshop may not be convenient.”*

[Staff 2]

*“I find that if it is going to be like a, um, how do you say that, like a face-to-face, I more like have difficulty in expressing myself, I guess. So, for having the Zoom, like with a Zoom, it make me, like, it’s more helpful on my side, I guess. You know, to think that I was, I’m going to say that I’m in my comfort zone as well, though, it makes me more relaxed. But I try, I try. I was speaking up so, yeah.”*

[Student 2]

##### Drawbacks of Zoom™—“Names with Black Boxes”

While Zoom™ had the benefit of encouraging participation, there were also drawbacks, as identified by the staff participants. There were two staff members that highlighted the importance of being able to visualise other people when engaging on Zoom™. Another issue raised by a staff member was that there was limited privacy with cameras being turned on. One student expected that the workshops would not be as engaging due to previous experiences with Zoom™ classes, and this workshop experience was different, which emphasises the importance of good facilitation when using Zoom™.

*“Zoom is impersonal lends itself to stilted conversations. A person in our room had their camera off and because I could not visualise her, a comment she made.”*

[Staff 5]

*“I think, as not a requirement, but just having, just putting faces to names, that’s really important. Like, talking to names with black boxes I don’t find that engaging.”*

[Staff 2]

*“Other issues that we’ve always encountered with, you know, student privacy, living in shared accommodation, or I don’t know how it would work with those other factors unless, you know, we were to provide them with a Zoom room on campus to say “here we go, Zoom is booked for you on campus” and then what’s the point of having Zoom if we’re going to have somebody come to campus. Why not come to campus?”*

[Staff 2]

*“Like I didn’t expect it would be that good with Zoom, ‘cause my previous Zoom classes, it’s not, like, that engaging.”*

[Student 3]

##### Reminders—“I’m Quite Forgetful”

While calendar invites were sent to all participants, the students found that the reminders sent to them alerting them of upcoming workshops motivated them and kept them interested. Students have many competing commitments, and engaging with them by providing reminders enabled them to participate; otherwise, they may have forgotten about the impending workshop.

*“I found it very helpful that you guys give us a email of the Zoom link just before it starts.”*

[Student 1]

*“I really liked how [organiser], she always kept on giving me reminders of in two weeks’ time I’m gonna have this, and she gave us a Zoom meeting and she was very nice in the way how she wrote the messages ‘cause I think reminders is good for me because, like I said before, I’m quite forgetful on what to do.”*

[Student 3]

#### 3.2.3. Category 3—Recruitment

A multimodal recruitment strategy was the most effective form of recruitment, as it enabled participants to engage via their preferred manner. The use of a video recording on the Blackboard learning site, email, and direct approach were seen as effective strategies for recruitment.

##### Video and Email Recruitment—“Oh, I Have to Participate in This”

One participant found the recruitment video engaging and expressed that it generated interest in the workshops; however, the most preferred method of recruitment was the use of email, with most participants responding to the email which was sent to them. The participatory nature of the workshops was an attraction for one participant, as they expressed surprise that the email was not a request to complete a survey, but rather to participate in the workshops.

*“there’s a video of you talking about the oral health thing. So, in my mind I was thinking ‘oh this is actually quite interesting’”*

[Student 3]

*“looking at the email before, it’s kind of like: “Oh, like, oral health”. Like, I thought, it would be only like [be a] survey or questionnaires and all of a sudden, like, “Oh, I have to participate in this one”*

[Student 2]

*“I responded to Facilitator’s email because, yeah, like everyone said, we tend to overlook oral health a lot in nursing”*

[Staff 1]

##### Direct Approach—“Facilitator Approached Me”

A direct approach to recruitment was adopted for two staff participants who had previously been involved in the larger project. Inclusion of participants who had previously been involved in the project was an effective recruitment strategy to employ, as the insight provided by these participants was useful in generating meaningful discussions.

*“I was introduced to the study through Facilitator actually. Facilitator asked me if I wanted to be a part of a project, and I started working with Facilitator last year I think”*

[Staff 2]

*“I think Facilitator approached me, what, must have been last year”*

[Staff 3]

#### 3.2.4. Category 4—Sensitising

To ensure that workshop participants were prepared for what would be covered in the workshops and to generate engagement and robust discussions, it was important to prepare the resources that would be effective to achieve this task. The booklet prepared was an effective resource, which was described as succinct and relevant by staff; however, a recommendation was given that it required clearer descriptions of the role and expectations for participants in the workshops.

##### Student and Staff Expectations—“It May Not Have Been as Clear to Others”

While the booklet was engaging and described Mezirow’s Transformative Learning Theory as well as the best practice learning activities, student participants felt that a clearer explanation of the expectations and their role in the workshops was required, as these two aspects caused some confusion. One staff member identified that while the booklet was a good resource, without prior knowledge of the project, the booklet may not have been clear on its own.

*“I thought it was pretty engaging. Like, she listed the theory, why it’s important for transformative learning, and then she had like Mezirow’s diagrams and all and then she listed the activities, and she gave like the description for those activities like what do they mean. So, I think that was really good. But, yeah, it was just like the understanding what **we** should do. That’s the only thing I was confused about.”*

[Student 3]

*“It was ok for me, but I have a little bit of knowledge about your project, it may not have been as clear to others, but facilitator did set it up well.”*

[Staff 5]

##### Preparation Time and Relevancy—“It Didn’t Take too Long to Prep”

The booklet was deemed by staff to be relevant, and the preparation time for the workshops was adequate. The information in the booklets prompted discussions in the workshops, and the participants expressed that minimal reading time was required to understand the concepts.

*“I found it was just enough to prompt you to think about stuff before you came into the meeting without, sort of, having lots and lots of to read over.”*

[Staff 1]

*“Yeah it was easy and you could just look at it and understand what you’re trying to say, like, it didn’t take too long to prep.”*

[Staff 4]

*“Very succinct. Um, and quite relevant.”*

[Staff 3]

#### 3.2.5. Category 5—Facilitation

Being able to facilitate robust discussions is essential in generating good codesign outcomes. The inclusion of all participants by the facilitator in the discussions was well received by participants. Both student and staff participants enjoyed the discussions that were held in the workshops, and innovative ideas were developed because of these discussions. The workshop groups consisted of a mix of staff and students, and while this was received well by staff, the students were a little more apprehensive.

##### Robust Discussions—“All of Us Were Given a Chance to Speak”

Both student and staff participants were satisfied with the robust discussions that were held in the workshops, with participants from different backgrounds expressing their ideas, sharing their experiences, and generating discussion. One staff member expressed that inclusion of the teaching staff from both the theoretical subject and the practical subject was a very good idea, as the process gave insight into the breadth of oral health education. To enable robust discussions, it was important that the facilitator included all participants in the discussion.

*“Discussion went really well. I enjoyed the process. Like all of us were given a chance to speak up. Like, no one, you know, like, you know was monopolising the discussion.”*

[Student 2]

*“Very engaging. Lots of great ideas. I was quite surprised, I think I told you, there was a particular student, and I was really surprised. I thought perhaps, you know, I was thinking perhaps the student has got some oral health background and it was really great, um, to see people from all sorts of backgrounds engaging with really good ideas.”*

[Staff 2]

##### Facilitation of the Discussions—“You Feel like You Are Included in the Discussion”

Participants expressed that the facilitator gave all participants an opportunity to express their ideas and thoughts to the group. Being inclusive was an effective form of facilitation. One participant recommended that they would have preferred it if, while the facilitator was writing down their ideas, what was being written down was visible to all in the group to keep track of the discussion. For instance, if the workshop is conducted online, using the screen share option on Zoom was suggested.

*“It would be nice to see what you were writing so you can keep track of the discussion, ‘cause some of the ideas people say I can forget or something like that, or I wouldn’t think that much about.”*

[Student 3]

*“Yeah, well I think when the facilitator takes the name of the person, like “Oh Student2, what do you think of this?” or “Student1, what do you think of this?” then it gets you more engaged. You feel like you are included in the discussion.”*

[Student 3]

One staff participant felt that more dynamic discussions could have been held if the participants had been face-to-face and able to write on whiteboards. In the case of conducting the workshops online, the use of the annotate feature on Zoom may have proven to be useful to facilitate the discussion in this way.

*“I just feel like we probably would have had a more, um, dynamic discussion if we were all sort of sitting together in one room and, perhaps, like writing on the board or something where you can really let things flow out.”*

[Staff 1]

##### Staff and Students Working Together—“These Guys Are Thinking Outside the Box”

Student participants were a little apprehensive about having staff in the same workshop, as it was felt that if there had been a prior conflict with a staff member that was present, this may have impacted their ability to participate. Another student participant felt a sense of slight intimidation with staff in the same workshop.

*“If I had any bias towards lecturers, or maybe if lecturers were present, I may have withheld some of the information. I don’t know like… maybe they’ll judge you and then they’ll mark you down.”*

[Student 3]

*“But just, kind of, that sense of intimidation.”*

[Student 1]

On the other hand, staff participants did not express displeasure with students being present; however, they realised that they may not be up to date with innovation. The staff overall were greatly impressed with the novel ideas that the students were presenting and felt that the students had expertise to share and were “forward thinkers” by “thinking outside the box”. Examples of these innovations include the use of a SWOT (Strengths, Weaknesses, Opportunities, Threats) analysis for the self-reflection and the use of animations, vlogs, podcasts, or vodcasts to deliver the content.

*“My thinking is very linear. Like when I think of curriculum, I know how I’m going to deliver it, but these guys are thinking outside the box. ‘Oh, we should do this, and we should do this’, I was like, what? I should know this stuff.”*

[Staff 2]

*“You forget that there are students out there that have a lot to contribute. And then when you come across them, it’s quite impressive and you think, well, I suppose the profession will be fine cause we do have students who are forward thinkers or think, just… think full stop.”*

[Staff 1]

*“But, it’s like everyone’s just said, it was amazing to see how much of an insight they have actually and we probably miss on those things when we rush into the two-hour classes and we are just trying to tell them everything that we know. Maybe we need to more often get back from what they know as well, because it’s been a wonderful experience. Yeah. Mind blowing.”*

[Staff 4]

## 4. Discussion

This study aimed to develop an oral healthcare educational intervention using the codesign framework described by Dietrich, Trischler, Schuster, and Rundle-Thiele [10] and to evaluate the codesign process. Resourcing effectively ensured that participants had a range of learning activities to choose, and effective planning and good facilitation supported inclusive practices, giving participants an equal voice. Overall codesign was a useful method to use to develop the oral healthcare educational intervention in this study, and on the whole, the process was successful.

An interesting finding in this study was the perceptions that students have regarding working with faculty staff to develop interventions. A degree of apprehension was reported by students in this study. While the study by Woods and Homer [16] with a codesign between staff and pre-arrival first-year students did not evaluate the students’ experiences of working with staff, they were able to produce codesigned learning activities in a manner similar to this study. Students’ perceptions in this study of perceived consequences of working with academics could be due to previous experiences with faculty staff within the classroom, including issues with staff commitment and rapport. Xiao and Wilkins [17] highlighted in their study on lecturer commitment that student satisfaction was determined by the level of commitment a lecturer had to their students. In addition to the study by Xiao and Wilkins [17], a study evaluating teaching from the perspective of students [18] identified that building lecturer–student rapport was very important, and while this study is dated, the concept remains relevant today. The dearth of literature surrounding this phenomenon highlights the need for further research on the topic.

During the COVID-19 pandemic, Zoom™ video communications became a popular communication method in a number of settings [19,20], with the codesign workshops in this study being no exception. In this study, it was identified that the convenience of Zoom™ workshops enabled participation, for both student and staff participants. In addition to this, Anene and Idiedo [19] highlighted that participating in Zoom™ workshops from the comfort of home and eliminating travel risks were other benefits that came from using Zoom™ as a platform for conducting workshops. An interesting finding in this study was how students perceived the workshops via Zoom™. One participant in this study did not think that the workshops would be engaging based on previous classroom experiences. However, this participant reported the codesign workshop experience to be engaging, which is supported by the experiences of Kent, George, Lindley, and Brock [20], whereby the online teaching workshops were considered engaging. These findings highlight that the quality of the facilitation of online workshops plays a key role in the engagement of participants and needs to be considered when planning for codesign workshops.

Building collaborative partnerships between service providers and consumers is a hallmark feature of codesign, and engaging students as partners in the codesign process provides students a voice in the development of curriculum [21]. Not only can students act as co-creators, but they also offer an expert voice [22]. This was demonstrated in this study, whereby faculty staff were impressed with the amount of insight students had and the contemporary ideas they had in the development of the oral healthcare educational intervention. While the piece by Cook-Sather et al. [23] discussed the integration of students in the publication process, they also highlighted the unique voice that students bring to co-creation and the importance of the expertise that students bring to a partnership. This is especially true in this study, whereby students were not only innovative in their approach but were contemporaneous with their ideas, adding richness to the experience of codesign as well as the discussions conducted in the workshops.

### 4.1. Strengths and Limitations

A limitation of this study is that the sample did not include men, as the only male participant that responded did not attend the first workshop. Another limitation of this study is that the sample size was small, particularly with student participants, thereby potentially lowering the representation of the entire student cohort. The implication of this is that the results generated in this study are specific to this study. Other studies may generate further insights, as different student cohorts may have differing needs and ideas. Thus, by considering the results of other studies, this would make the process transferable across different settings based on need, which is a strength of this study. Another strength of this study was that the approach to codesign was novel and involved using a learning theory to underpin the study and a codesign framework to develop the intervention. Additionally, this study could be used by other researchers and educators to serve as a blueprint for developing educational interventions.

### 4.2. Future Areas of Research

There is a dearth of research on the evaluation of codesign processes within higher education. Further research in this area may assist researchers in the planning and undertaking of codesign in a manner which is meaningful. Another area of further research is to evaluate the effectiveness of the oral healthcare intervention developed in this codesign process through a pre–post-test study.

## 5. Conclusions

Employment of a codesign approach was beneficial in the development of an oral healthcare intervention. Good preparation, planning, and facilitation achieved successful outcomes from the codesign process. The use of the six-step process allowed both staff and students to engage with the material and participate in rich, meaningful conversations. Each participant brought their unique experiences and expertise to the discussion, which led to collaboration between nursing students and faculty in the development of a novel oral healthcare educational intervention within an Australian nursing education context.

## Figures and Tables

**Table 1:** Final codesigned learning intervention.

Mezirow’s Transformative Learning Theory Phase	Learning Activity	Time for Activity
Disorientating dilemma	Case study with no diagnosis—picture of a patient with periodontitis (theoretical subject).	4 min
2. Self-examination and (self-reflection)	1 min essay (theoretical subject).	1 min
3. Critical assessment of assumptions (reflection with others)	Class discussion (theoretical subject).	5 min
4. Plan a course of action	Student self-initiates a course of action which will allow the student to identify gaps in learning and ways in which to bridge these gaps.	Self-directed; however, there was a one-week gap between theoretical and practical classes.
5. Acquire new knowledge	Subject-matter-expert-led podcasts (short duration) (theoretical subject).	6 min
	2. Dental models (practical subject).	Unlimited time
	3. Video demonstration and tutor demonstration if requested by student of an oral health assessment (practical subject).	4 min
	4. Picture guide (normal and abnormal pathology) (practical subject).	Unlimited time
	5. Practice quizzes (theoretical subject).	10 min
6. Exploring and trying new roles	Using the Oral Health Assessment Tool, students practice oral health assessment in class and in clinical settings with facilitator (non-assessable) (practical subject and clinical placement).	15 min
7. Becoming confident and competent	Continued practice (clinical placement).	No time limit

**Table 2:** Categories and sub-categories.

Category	Sub-Category
Category 1—Resourcing	1.1 Learning activities—*“catalyst to make me start thinking”* 1.2 Learning activities—*“triggers that critical thinking”*
Category 2—Planning	2.1 Workshop timing—*“I didn’t realise we went over”* 2.2 Benefits of Zoom™—*“convenience definitely”* 2.3 Drawbacks of Zoom™—*“names with black boxes”* 2.4 Reminders—*“I’m quite forgetful”*
Category 3—Recruitment	3.1 Video and email recruitment—*“Oh, I have to participate in this”* 3.2 Direct approach—*“facilitator approached me”*
Category 4—Sensitising	4.1 Student and staff expectations—*“it may not have been as clear to others”* 4.2 Preparation time and relevancy—*“it didn’t take too long to prep”*
Category 5—Facilitation	5.1 Robust discussions—*“all of us were given a chance to speak”* 5.2 Facilitation of discussions—*“you feel like you are included in the discussion”* 5.3 Staff and students working together—*“these guys are thinking outside the box”*

## Data Availability

Not applicable.

## Supplementary Materials

The following supporting information can be downloaded at: https://www.mdpi.com/article/10.3390/ijerph20064919/s1, Supplementary Document S1: Participant Codesign Booklet.
