# Supplementary material for: Using Codesign to Develop a Novel Oral Healthcare Educational Intervention for Undergraduate Nursing Students
Source: Int J Environ Res Public Health. 2023 Mar 10;20(6):4919. doi: 10.3390/ijerph20064919 (PMC10048837; doi:10.3390/ijerph20064919)
Supplement: Supplementary file 1 [file ijerph-20-04919-s001.zip › ijerph-2250664-supplementary.pdf]

Oral Health Co-design

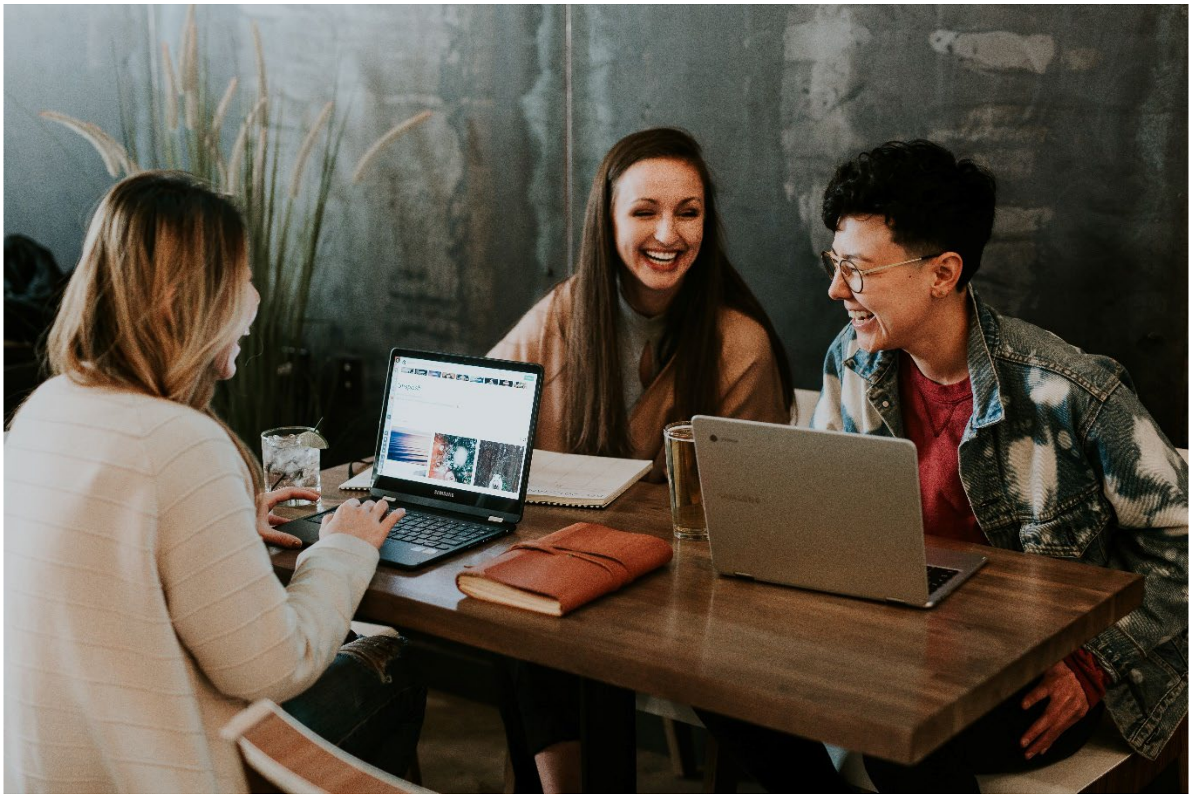

Photo by Brooke Cagle on Unsplash

Western Sydney University
School of Nursing and Midwifery
2021

What is Co-design and What to Expect at the Workshops

Co-design

Co-design is an approach that is used to facilitate the development of learning materials between students and teaching staff. It provides participants an opportunity to discuss, collaborate and contribute to solving real-world problems.

The goal of the workshops is to develop a module of learning activities that will be implemented across both HV2 and PPE4 related to oral healthcare.

At the first workshop, you will be divided into groups which will consist of both students and teaching staff. Guided by an example which follows Mezirow's Transformative Learning Theory, you will develop the activities. This is your chance to be creative!

At the second workshop, which will be held approximately a week after the first, your group will present the learning activities which you have all developed. After the presentations, we will break off into staff and student groups, and undertake the evaluation of the workshop process in the development of the education module.

Photo by Joel Muniz on Unsplash

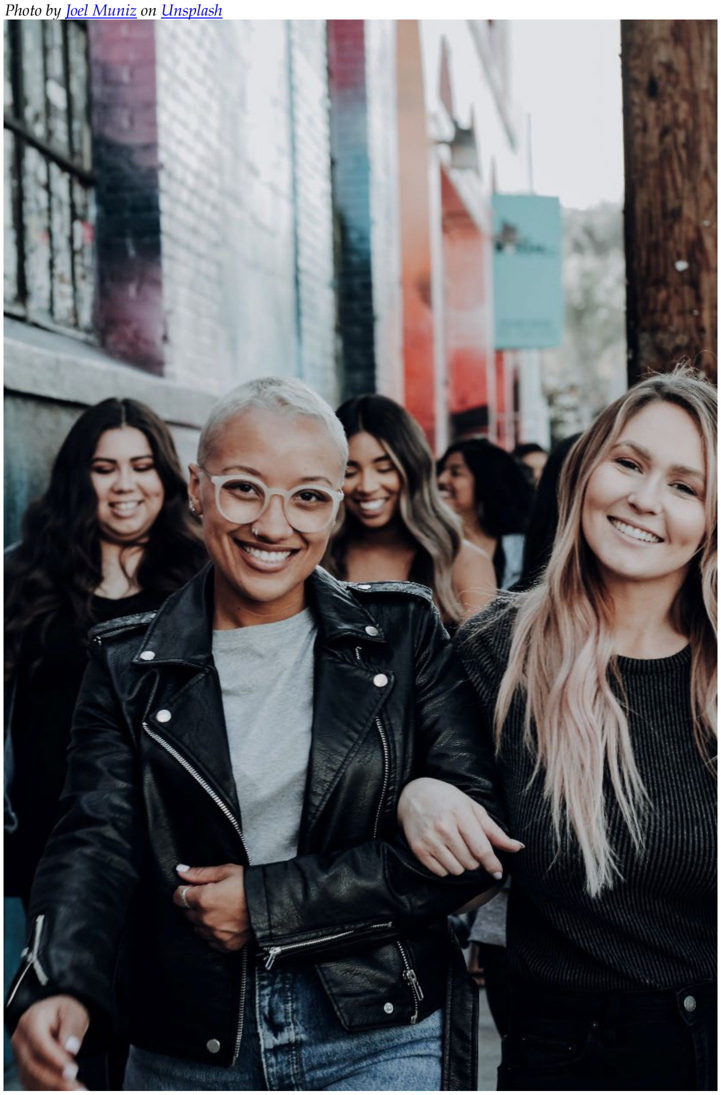

Let's Transform…

Mezirow's Transformative Learning Theory

Using this seven-phase theory, students will first be presented with a **disorientating dilemma** (something that will make them question what they know). Next, is the phase of **self-examination** and **critical assessment of assumptions** by discussing with others about this dilemma. When the learner realises the need of more knowledge or skills, they **plan a course of action** and seek to **acquire this new knowledge** through various methods, for example, through self-directed learning or material provided as part of the coursework. Following the phase where students are equipped with new knowledge, they will then **try new roles** and practise their newly acquired skills until they **are confident and competent**.

Suggested Learning Activities

Consider an activity for each phase of Mezirow's theory

Disorientating Dilemma

Reverse case study
1-minute essay
Case study with no diagnosis

Self-examination

1-minute essay
Reflective writing piece

Assessment of assumptions

Group discussion
Class discussion
Think-pair-share

Acquiring new knowledge

Embed learning material in lecture
Tutorial pre-work / online modules
Literature
Picture guide and dental models in CPU
Video demonstration
Demonstration by tutor

Trying new roles

Oral health assessment practice in CPU
Practice on clinical placement

What are these learning activities?

Reverse case study

In this activity, the student is given the name of a medication and a sentence or two that sets the scene. Based on the medication they are provided; the student then needs to write the case study information, which includes the story, clinical manifestations, observations and management. The student can then take this case study from HV2 to PPE4.

1-minute essay

In this activity, the student is asked to write all they know about oral health in 3 minutes. This activity is a good reflective tool.

Case study with no diagnosis

In this activity, the student is given the details of the case study, however there is no diagnosis of the condition. The student then needs to work towards finding out what is wrong with the patient. The student can then take this case study from HV2 to PPE4.

Reflective writing piece

This activity involves a short written reflection on the student's own understanding of oral health concepts. It is estimated that this activity will run for no more than 5 minutes.

Photo by Štefan Štefančík on Unsplash

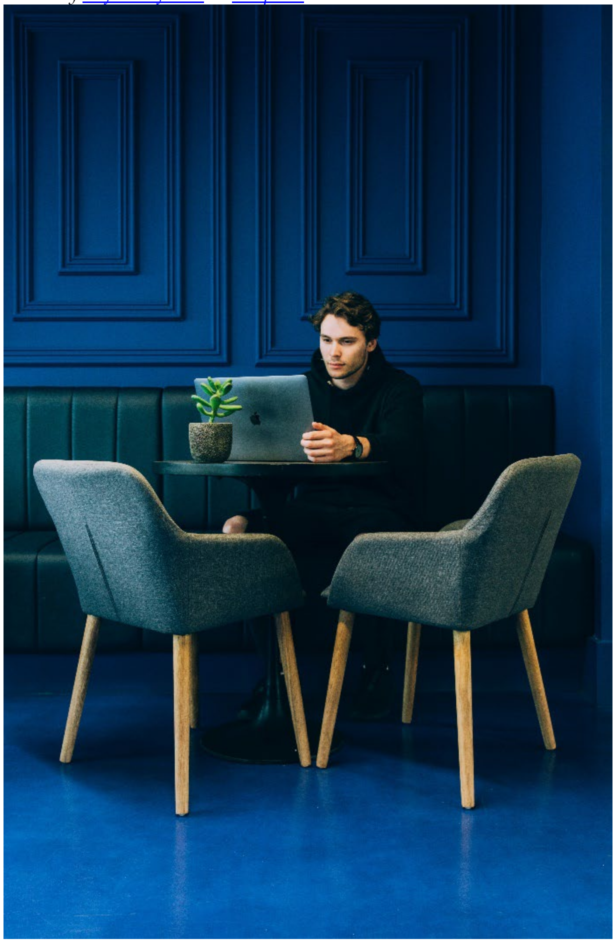

Group discussion

This activity is a discussion held within a small group of students. The tutor may facilitate this discussion; however, it is led by the students.

Class discussion

This activity is a discussion held with the entire class. The tutor facilitates this discussion as students contribute and lead the discussion.

Think-pair-share

In this activity, the students are given an oral health concept/question and asked to think about the concept/question and develop a response. The students are then divided into pairs and asked to discuss their responses with each other.

Embed learning material in lecture

The lecture material will have added oral health material to enable this as a resource that students can use.

Tutorial pre-work / online modules

This activity would see the students engaging and completing pre-set material prior to coming to class. This can be in the form of online learning activities.

Literature

This activity consists of the Unit Coordinator providing the students with current oral health readings.

Photo by kraken images on Unsplash

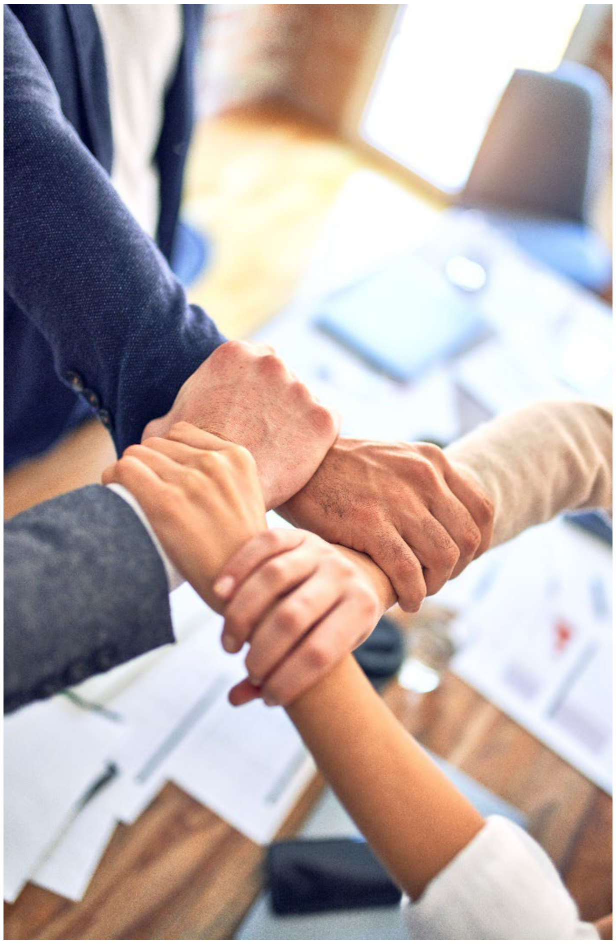

Picture guides and dental models in the CPU

To assist in building understanding of concepts, a resource guide consisting of images of oral disease and dental models demonstrating these diseases also will be available for students to use.

Video demonstration

A video demonstration of an oral health assessment, using the Oral Health Assessment Tool, will be available to view prior to the CPU.

Demonstration by tutor

In this activity, the oral health assessment will be demonstrated by the tutor.

Oral health assessment practice in the CPU

Students will have the opportunity to practice the oral health assessment in class.

Practice on clinical placement

Students will be able to develop confidence and competence while practising on clinical placement.

Photo by LinkedIn Sales Solutions on Unsplash

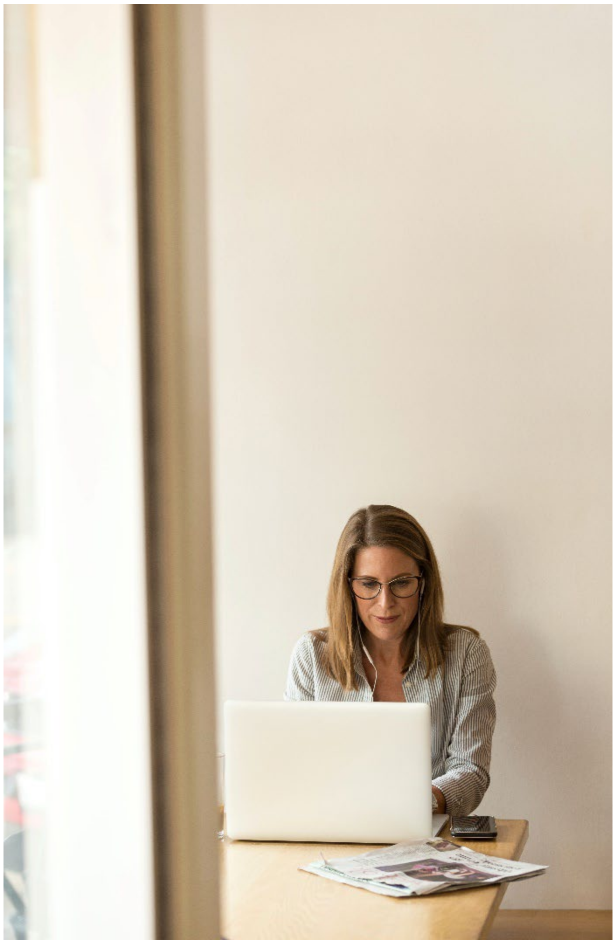

For example:

A reverse case study is presented to the students in HV2 giving them 1 medications (analgesia) that should lead to think about a dental caries (**disorientating dilemma**). The students are then asked to think-pair-share about all they know about oral health (**self-examination and assessment of assumptions**). Students will then be advised that they are to take the reverse case study to their PPE4 class. As students may be disorientated about the reverse case study, they will go and **acquire new knowledge** by accessing pre-tutorial work, lecture material and readings available on vUWS as well as a video demonstrating an oral health assessment. In the CPU, the students are now equipped to take on their **new role** by practicing an oral health assessment guided by a picture guide of different oral health conditions and supplemented by dental models. At the completion of the CPU the students are now equipped to further practice their skills while on clinical placement.
